# Higher resting metabolic rate in long-lived breeding Ansell’s mole-rats (*Fukomys anselli*)

**DOI:** 10.1186/s12983-017-0229-6

**Published:** 2017-09-22

**Authors:** Charlotte Katharina Maria Schielke, Hynek Burda, Yoshiyuki Henning, Jan Okrouhlík, Sabine Begall

**Affiliations:** 1 Faculty of Biology, University of Duisburg-Essen, Essen, Germany; 2 Faculty of Forestry and Wood Sciences, Czech University of Life Sciences, Praha, Czech Republic; 3 Faculty of Science, University of South Bohemia, České Budějovice, Czech Republic

**Keywords:** Aging, Reproduction, Mole-rat, Resting metabolic rate, Oxidative stress

## Abstract

**Background:**

Reproduction is an energetically expensive process that supposedly impairs somatic integrity in the long term, because resources are limited and have to be allocated between reproduction and somatic maintenance, as predicted by the life history trade-off model. The consequence of reduced investment in somatic maintenance is a gradual deterioration of function, i.e. senescence. However, this classical trade-off model gets challenged by an increasing number of contradicting studies. Here we report about an animal model, which adds more complexity to the ongoing debate. Ansell’s mole-rats are long-lived social subterranean rodents with only the founder pair reproducing, while most of their offspring remain in the parental burrow system and do not breed. Despite of a clear reproductive trade-off, breeders live up to twice as long as non-breeders, a unique feature amongst mammals.

**Methods:**

We investigated mass-specific resting metabolic rates (msRMR) of breeders and non-breeders to gain information about the physiological basis underlying the reproduction-associated longevity in Ansell’s mole-rats. We assessed the thermoneutral zone (TNZ) for breeders and non-breeders separately by means of indirect calorimetry. We applied generalized linear mixed-effects models for repeated measurements using the msRMR in the respective TNZs.

**Results:**

TNZ differed between reproductive and non-reproductive Ansell’s mole-rats. Contrary to classical aging models, the shorter-lived non-breeders had significantly lower msRMR within the thermoneutral zone compared to breeders.

**Conclusion:**

This is the first study reporting a positive correlation between msRMR and lifespan based on reproductive status. Our finding contradicts common aging theories, but supports recently introduced models which do not necessarily link reproductive trade-offs to lifespan reduction.

## Background

Aging is defined as a gradual decline in intrinsic physiological function leading to an increase in morbidity and mortality rate (reviewed in: [1]). However, the mechanisms behind aging, i.e. senescence processes are still poorly understood. The disposable soma theory is a prevailing model of aging, which is based on a trade-off between energy demanding processes, including growth, somatic maintenance, and reproduction due to limited resource availability [2]. Reproduction is energetically expensive [3–6], and is often considered a central force shaping different life histories [7]. As soon as an animal starts reproducing, energy resources are allocated to reproduction. Consequently, less energy is available for somatic maintenance and protection, leading to a gradual accumulation of somatic damage. This process is thought to be even amplified, because reproduction increases the metabolic rate to cover the increased energy demand [8]. This increase in metabolic rate is predicted to result in oxygen radicals, i.e. reactive oxygen species (ROS), highly reactive byproducts which cause oxidative damage to DNA, lipids and proteins [1].

However, high energy turnover does not necessarily increase oxidative damage and mortality. Contrary to earlier expectations, correlational and experimental studies published recently show no negative effect of high metabolic rate on lifespan [9–12], or even a positive association [13]. Moreover, in the brown trout, higher metabolic rates were negatively correlated with levels of H_2_O_2_, a highly potent ROS. These controversial associations between metabolic rate and oxidative damage and / or lifespan can in parts be ascribed to different experimental setups and tissues studied (reviewed in: [8, 14, 15]). Moreover, some studies indicate that at least reproductive females have an increased ability to protect from oxidative damage, termed oxidative shielding, in order to protect the offspring from prenatal somatic damage (reviewed in: [8]). Consequently, more research is needed to gather representative data from animals with different life histories, to gain a comprehensive understanding of how life history trade-offs influence lifespan. Here we present the data of a subterranean mammal with a unique life history to contribute to the discussion stated above.

Ansell’s mole-rats (*Fukomys anselli*) are subterranean rodents of the family Bathyergidae with an extraordinary long lifespan (22 years being the maximum recorded age thus far; own observations). They live in multigenerational families where typically only the founder pair (breeders) reproduces. Most of the offspring (non-breeders) forego reproduction and remain in the natal family. Incestuous mating (i.e. between brothers and sisters) usually does not occur, however, adult non-breeders readily mate with unrelated conspecifics if given a possibility [16, 17]. A clear contradiction to the classic trade-off model has been shown in this species: breeding individuals live up to twice as long as their non-breeding counterparts, a feature which is unique amongst mammals [18].

Thus far, proximate factors contributing to this bimodal aging pattern are not known. In contrast to naked mole-rats where reproductive behavior of non-breeders is aggressively suppressed by the mother, Ansell’s mole-rats facilitate incest avoidance by individual recognition. Moreover, they exhibit pronounced sociopositive behaviors like grooming and huddling between all family members [19]. Moreover, previous studies showed that daily activity between breeders and non-breeders does not show differences, and social rank does not influence life expectancy [18, 20, 21]. Hence, extrinsic factors like aggression, fighting and higher workload in non-breeders are not likely to influence the lifespan difference in first-place. Thyroid hormone levels, as a possible intrinsic factor, showed no status-dependent difference, as well [22].

Here, we test the hypothesis that breeders and non-breeders of Ansell’s mole-rats differ in their mass specific resting metabolic rate (msRMR), as a possible approach to understand the bimodal aging pattern.

## Methods

### Study animals

We measured oxygen consumption (VO_2_) using open flow respirometry in 26 Ansell’s mole-rats (six reproductive females and six reproductive males, eight non-reproductive females and six non-reproductive males) in order to determine msRMR for both reproductive states (Table 1). None of the reproductive females were pregnant or lactating during the time of measurements. Of the 26 tested Ansell mole-rats, four non-reproductive (two males, two females) and four reproductive animals (three males, one female) were wild-captured and lived for more than one year in captivity, thus being fully acclimatized to laboratory conditions. Trapping and export of wild Ansell’s mole-rats were approved by the Zambian Wildlife Authorities (permit numbers 4790 and 4060, issued June 7th, 2010). Maintenance was approved by the Veterinary Office of the City of Essen (AZ: 32-2-1180-71/328). Housing conditions have been described elsewhere [23]. Briefly, mole-rats were kept on animal litter in glass terraria in the animal facilities of the Department of General Zoology at the University of Duisburg-Essen (Germany). Mole-rats were fed ad libitum with carrots, potatoes, and once per week with apples, salad and cereals. Light conditions were 12D:12L. All experiments were performed in accordance with the guidelines and regulations of the local authorities. All experiments were approved by the North Rhine-Westphalia State Environment Agency (permit no. AZ: 87-51.04.2010.A359/01) and have been performed in accordance with their guidelines and regulations.

**Table 1:** Basic parameters and mean mass-specific resting metabolic rate (msRMR) of *Fukomys anselli*

Sex	Status	N	Age (years)	Body mass (g)	Range of msRMR (ml O_2_ × g^−1^ × h^−1^)	Mean msRMR (ml O_2_ × g^−1^ × h^−1^)
M	R	6	6.9 ± 2.4	83.9 ± 10.5	0.77–1.71	1.17 ± 0.13
	NR	6	4.8 ± 2.8	108.8 ± 21.4	0.43–1.46	0.91 ± 0.17
F	R	6	8.9 ± 3.1	87.2 ± 21.8	0.63–1.80	1.17 ± 0.20
	NR	8	2.4 ± 1.2	68.2 ± 8.9	0.42–1.79	0.86 ± 0.21
Grand mean		26	5.8 ± 4.3	85.6 ± 21.4	0.42-1.80	1.02 ± 0.23

Age, body mass and mass-specific resting metabolic rates within the boundaries of their respective thermoneutral zones (non-reproductive animals: 26–30 °C; reproductive animals: 28–33 °C) of studied *Fukomys anselli* (*N* sample size, *M* males, *F* females, *R* reproductive; *NR* non-reproductive). Values are given as mean ± SD

### Experimental procedure

We first determined the thermoneutral zone (TNZ) for both reproductive states by measuring oxygen consumption (VO_2_) of Ansell’s mole-rats at 13 different ambient temperatures (T_a_) ranging between 10 and 40 °C. For non-reproductive individuals the sample size was 14 at temperatures 10, 15, 20, 25, 26, 28, 30, 32, 33, 34, 35, and 37 °C and 11 at 40 °C. For reproductive animals, the sample size was 12 for temperatures 28, 30, 32, and 33 °C and 4 for temperatures 20, 25, 26, 34, 35, and 37 °C. The tested temperatures did not include extremes for reproductive animals in order to avoid impairments of reproductive animals from established colonies due to hypothermia and/or hyperthermia. Especially high temperatures can be critical, as prolonged exposure of Ansell’s mole-rats to ambient temperatures about 42 °C were shown to be potentially lethal (previous own accidental observations).

Before each experimental trial, the animals were food deprived for at least 12 h. To establish an oxygen analyzer baseline, the oxygen analyzer (Servomex Type 5200 Multi Purpose, Crowborough, UK) was calibrated using 99.999% N_2_ (Air Liquide, Düsseldorf, Germany) for 0% oxygen and compressed outdoor air for 20.95% oxygen at the beginning and at the end of each experimental trial. After initial calibration, the animals were placed in a custom-made tight stainless steel metabolic chamber with a volume of 896 ml (16 cm × 7 cm × 8 cm) supplemented with tissue paper as nest litter. The chamber was closed with a clear Perspex lid to enable direct observation of the animal’s behavior inside the chamber. The watertight chamber was submerged in a water bath (temperature controlled by HAAKE D1, Type 001–3603, Germany). The animals spent at least 30 min within the chamber to acclimatize before measurements started. Measurements were finished when the animal calmed down within the chamber and stable data were gathered over a period of 10 min, which can be considered a truly resting state. In this state, the animals lay down and breathe regularly. If an animal did not calm down within 60 min, the experiment was stopped and repeated later with a time lag of three days at minimum. Under extreme temperatures (<15 °C and >35 °C) the maximum duration of an experimental trial was reduced to 30 min to avoid hypothermia or overheating, respectively. Temperature within the chamber was measured with a digital thermometer (GMH 3230, Greisinger Electronic, Germany) with its probe placed behind a grid to avoid damage by the animals. Ambient air was pushed through the chamber at a constant rate of 350 ml × min^−1^. The incurrent airflow was regulated by a flow controller (Model 35830, Analyt-MTC, Müllheim, Germany), following a carbon dioxide (Sodalime) and a water trap (indicating Drierite) before the oxygen content was measured by a paramagnetic oxygen sensor (Servomex Type 5200 Multi Purpose, Crowborough, UK). Software DIAdem 8.0 (National Instruments, Germany) was used to visualize and to record the oxygen content of the excurrent air every second. Immediately after each trial the animals were weighted. Oxygen consumption was calculated following the equation of Lighton [24]:

$$ V{O}_2=\frac{F_{ri}\left({F}_i{O}_2-{F}_e{O}_2\right)}{1-{F}_e{O}_2} $$

with: VO_2_ = oxygen consumption (ml O_2_ × min^−1^), F_ri_ = incurrent flow rate (ml × h^−1^) F_i_O_2_ = oxygen incurrent fractional concentration (%), F_e_O_2_ = oxygen excurrent fractional concentration. Data gathered by the oxygen sensor were corrected to standard temperature and pressure conditions (273.15 K, 101.325 kPa). The RMR was calculated as a 10-min mean of lowest oxygen consumption and expressed in ml O_2_ × h^−1^. msRMR was then calculated as RMR divided by the individual’s weight and expressed in ml O_2_ × g^−1^ × h^−1^.

### Statistical analysis

The extent of TNZ was assessed separately for reproductive and non-reproductive Ansell’s mole-rats by a step-down procedure [25] of permutation version of the Jonckheere-Terpstra test [26]. Briefly, starting from the temperature, where the mean msRMR was the lowest, we tried to detect an increasing trend in msRMR data assorted by increasing ambient temperature. If a trend was detected, the msRMR data for the highest temperature were excluded and tested again until no trend in msRMR data was detected and the upper critical temperature was identified. The determination of the lower critical temperature was similar, but here the lowest temperature was excluded stepwise until no trend could be detected. The metabolic rate data were included in TNZ estimation only when the sample size for the given temperature comprised five or more values. The analyses revealed different ranges of TNZ for non-reproductive and reproductive individuals. The observed range of TNZ was 26–30 °C in non-reproductive and 28–33 °C in reproductive Ansell’s mole-rats, respectively (Table 2).

**Table 2:** Critical temperatures and Jonckheere-Terpstra statistical analysis for reproductive and non-reproductive *Fukomys anselli*

Parameter	Reproductive	Non-reproductive
Upper critical temperature	≥33 °C^a^	30 °C
Temperature range /JT/p	N/A	28...32 °C/368/0.0486
Temperature range /JT/p	32...33 °C/90/0.16	28...30 °C/102/0.43
Lower critical temperature	28 °C	26 °C
Temperature range /JT/p	26...33 °C/421/0.0002	25...30 °C/471/0.043
Temperature range /JT/p	28...33 °C/410/0.346	26...30 °C/246/0.1364

Upper critical temperature and lower critical temperature together with parameters of statistical analysis in reproductive and non-reproductive *F. anselli*. ^a^minimal value only, because of lack of RMR data for reproductive animals at temperatures above 33 °C, see Methods for more detail. JT represents Jonckheere-Terpstra-statistics, p is the probability of trend in data

To estimate the effects of various predictors on msRMR (dependent variable) within the TNZ boundaries, data were analyzed with generalized linear mixed-effects models for repeated measurements using the lme4 package [27] and Gamma distribution was assumed (link identity). First, we extended the null model to include one of the independent variables (square-root transformed body mass, ambient temperature as factor, reproductive status) and tested which of them optimally improved the model using the Akaike information criterion and model comparison by χ^2^-statistic. Subsequently, we extended this model by inclusion of different variables in the same way as described above and we continued until there was no variable left which would significantly improve the model (forward selection). Finally, interaction model of all significant factors was constructed. Individual identity was treated as a random factor. Since the age of some individuals was not known, the same procedure as described above with an additional factor age was applied to all animals with known age. This analysis showed that age is not a significant factor (results not shown). All calculations and statistical analyses were conducted using R 3.0.2 [28].

## Results

**Fig. 1:**
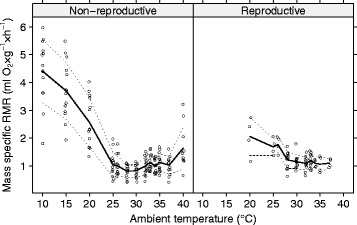
Mass specific resting metabolic rate of non-reproductive (left) and reproductive (right) *Fukomys anselli* at different ambient temperatures. Means are connected by a solid line, interrupted lines connect mean ± SD

**Fig. 2:**
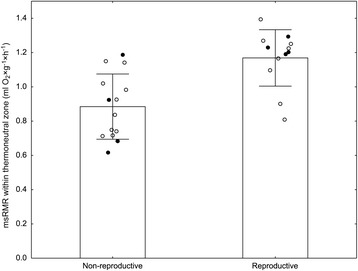
Mass specific resting metabolic rate (msRMR) of non-reproductive (*N* = 14) and reproductive (*N* = 12) *Fukomys anselli* within the boundaries of their respective thermoneutral zones (non-reproductive: 26–30 °C; reproductive: 28–33 °C). The mean msRMRs of each individual (open circles) throughout its TNZ are presented together with the mean of the respective reproductive status (bars) and its SD (whiskers). Full symbols indicate wild-derived animals

msRMR at different temperatures are depicted in Fig. 1. Both, TNZ and mean msRMR differed between reproductive and non-reproductive Ansell’s mole-rats (Tables 1 and 2). The mean msRMR of reproductive Ansell’s mole-rats (1.17 ± 0.17 ml O_2_ × g^−1^ × h^−1^) within their TNZ of 28–33 °C was significantly higher than the mean msRMR of non-reproductive animals (0.89 ± 0.19 ml O_2_ × g^−1^ × h^−1^) within their TNZ of 26–30 °C (Fig. 2, Table 1). The lowest mean msRMR (0.81 ± 0.23 ml and 1.1 ± 0.23 O_2_ × g^−1^ × h^−1^) was observed at ambient temperatures of 28 °C and 32 °C in non-reproductive and reproductive mole-rats, respectively. The optimal model of msRMR determined by generalized linear mixed models for repeated measurements consisted only of the reproductive status (AIC = 8.83; comparison with null model χ^2^ = 6.72, df = 1, *p* = 0.01) and showed fixed effects of 0.89 ± 0.07 and 1.17 ± 0.09 for non-reproductive and reproductive factor level, respectively. The difference itself (0.28 ± 0.09) is statistically significant (*t* = 2.995, *p* < 0.003) suggesting that the msRMR of reproductive individuals is about 30% higher than that of non-reproductive ones.

## Discussion

### Metabolic rate with respect to reproduction

Low msRMR is a common trait in bathyergid rodents interpreted as an ecophysiological adaptation to the subterranean habitat [29], and our measurements generally confirm previous studies. However, our finding that long-lived breeders of *F.* anselli have higher metabolic rates compared to shorter-lived non-breeders is novel. The higher metabolic rate observed in breeders is not surprising per se, since reproduction is energy demanding, especially for lactating females [8, 30, 31], albeit none of the reproductive females were pregnant or lactating during the time of measurements. In Damaraland mole-rats, breeding females also live significantly longer than non-breeding females [32], but no significant differences in msRMR of female breeders and non-breeding individuals were found. However, changes in daily energy expenditure were observed depending on seasonal changes in rainfall [33]. Higher msRMR in breeders compared to non-breeders of *F. anselli* are in line with findings in pregnant naked mole-rats, another long-lived bathyergid species (>30 years), which have a higher body temperature compared to their non-breeding counterparts [34]. To the best of our knowledge, metabolic data of reproductive naked mole-rats is not available, but higher body temperature suggests a higher metabolic rate. This assumption is also in line with most mammals, where energy intake and/or daily energy expenditure was shown to be increased in lactating females [35]. This aspect is most interesting since investment in reproduction was long thought to impair somatic maintenance according to the classical trade-off model, but recent findings refer to the trade-off model as being too simplistic [14]. Especially in terms of female reproduction, a meta-analysis from different homeothermic vertebrates by Blount et al. [8] has shown that in intraspecific comparisons between breeders and non-breeders, breeders had lower levels of oxidative damage in certain tissues. This effect could be attributed to upregulation of antioxidant defense mechanisms, such as glutathione or superoxide dismutase activity, which shows a tissue-dependent upregulation in several species during reproduction [36–39]. This oxidative shielding hypothesis, even if not consistent across different studies (reviewed in: [8]), suggests a reproduction-induced protection of mothers and offspring. Ansell’s mole-rats are continuously reproducing once they achieve the reproductive status. Oxidative shielding might protect the animals from detrimental pregnancy effects due to a higher energy turnover in female breeders compared to non-breeders. However, the bimodal lifespan in Ansell’s mole-rats is not sex-dependent, indicating a general effect in terms of reproductive status, msRMR, and lifespan rather than just a pregnancy effect restricted to females.

The mechanisms underlying the higher msRMR in male Ansell’s mole-rat breeders which do not lactate cannot be derived from the present results. Even so, it could be argued that reproduction in monogamous species includes parental care in male and female, thus it could be that a closer look at monogamous male breeders reveals a higher energy turnover during the reproductive phase as well. Furthermore, breeders are sexually active throughout the year, which could increase the overall msRMR in these individuals.

### Higher msRMR in long-lived breeders is not in line with classical aging theories

Oxidative stress as a main factor contributing to life history trade-offs is getting challenged by increasing contradictory studies [14]. Hence, higher msRMR in breeders is in line with those studies, which did not find any correlation [9] or even support a positive correlation between RMR and lifespan [11, 13, 40–43]. In a large comparative study by de Magalhães et al. [9], data from 300 mammalian species were analyzed, but after correcting for body mass and phylogeny, no influence of metabolic rate on lifespan, at least in eutherians, could be found. On the other hand, the developmental schedule of a species, i.e. age of sexual maturity and postnatal growth rate, were both correlated with longevity. Mole-rats have a relatively long gestational period of about 3 months (100 days), slow postnatal growth rates, and reach sexual maturity at about 1 year of age [44, 45]. These features are in line with developmental schedule-associated implications reported in de Magalhães et al. [9]. Although these developmental features might account for overall longevity in mole-rats, the distinct intraspecific lifespan difference between breeding and non-breeding Ansell’s mole-rats is still a special case which must be discussed in light of studies that support a positive correlation between msRMR and lifespan [18].

The uncoupling-to-survive hypothesis [46] complements simplistic theories of senescence by explaining apparent exceptions. It suggests that elevated oxygen consumption, a measure for msRMR in the present study, could be also observed due to uncoupling of proton flux in the mitochondria. This process, also referred to as inducible proton-leak, is facilitated by uncoupling proteins and increases RMR. On the other hand, inducible proton-leak is known to reduce ROS production by reducing mitochondrial membrane potentials [47]. Hence the higher msRMR measured in breeders of Ansell’s mole-rats could be due to higher rates of mitochondrial uncoupling compared to non-breeders. Several studies found higher rates of uncoupling in those laboratory mice that lived longer compared to other individuals with shorter lifespans [13, 41, 42]. A recent study even showed that mitochondrial H_2_O_2_ levels (which is a ROS) were negatively correlated to the metabolic rate in the brown trout [43]. However, in case of mole-rats this model should be considered carefully, since in naked mole-rats, surprisingly high levels of oxidative damage to DNA, lipids and proteins were found, which contrasts with the proposed benefit of mitochondrial uncoupling [48]. Nevertheless, it would be interesting to establish a protocol to investigate mitochondrial ROS in *F. anselli* in future studies. Mitochondrial uncoupling should be taken into account as a possible proximate mechanism contributing to the bimodal lifespan of *F. anselli*, but the adaptive background of such a selective protective mechanism on species level is still puzzling. It may be that the upregulation of protective, or in general, repair mechanisms are just a side effect of sexual activity in this species. For instance, long-lasting pair bonding could lead to higher oxytocin levels, known to decrease stress hormones and promote immunity (reviewed in [49]). Hence, further research should illuminate the linkage between different physiological processes, and how the different processes impacts an animal’s lifespan.

## Conclusions

Bathyergid species with their exceptionally long lifespan are already interesting animal models in current aging research, but this is the first study to report a positive correlation between msRMR and lifespan based on reproductive status. The bimodal lifespan of our animal model, the Ansell’s mole-rat, provides an exceptional opportunity to investigate protective mechanisms such as oxidative shielding on species level without the usual shortcomings related to experimental manipulation of the study animals. In general, our finding stresses the complexity of currently discussed aging mechanisms.

## References

[1] Höhn A, Weber D, Jung T, Ott C, Hugo M, Kochlik B, Kehm R, Konig J, Grune T, Castro JP (2017). Happily (n)ever after: aging in the context of oxidative stress, proteostasis loss and cellular senescence. Redox Biol.

[2] Kirkwood TBL, Holliday R (1979). The evolution of ageing and longevity. Proc R Soc Lond B Biol Sci.

[3] Deerenberg C, Pen I, Dijkstra C, Arkies B-J, Visser GH, Daan S (1995). Parental energy expenditure in relation to manipulated brood size in the European kestrel *Falco tinnunculus*. ZACS.

[4] McNab BK (2006). The energetics of reproduction in endotherms and its implication for their conservation. Integr Comp Biol.

[5] Speakman JR (2008). The physiological costs of reproduction in small mammals. Philos Trans R Soc Lond Ser B Biol Sci.

[6] Heldstab SA, van Schaik CP, Isler K (2017). Getting fat or getting help? How female mammals cope with energetic constraints on reproduction. Front Zool.

[7] Kirkwood TBL (2008). Understanding ageing from an evolutionary perspective. J Intern Med.

[8] Blount JD, Vitikainen EIK, Stott I, Cant MA (2016). Oxidative shielding and the cost of reproduction. Biol Rev.

[9] de Magalhaes JP, Costa J, Church GM (2007). An analysis of the relationship between metabolism, developmental schedules, and longevity using phylogenetic independent contrasts. J Gerontol A Biol Sci Med Sci.

[10] Furness LJ, Speakman JR (2008). Energetics and longevity in birds. Age.

[11] Munshi-South J, Wilkinson GS (2010). Bats and birds: exceptional longevity despite high metabolic rates. Ageing Res Rev.

[12] Selman C, McLaren JS, Collins AR, Duthie GG, Speakman JR (2008). The impact of experimentally elevated energy expenditure on oxidative stress and lifespan in the short-tailed field vole *Microtus agrestis*. Proc Biol Sci.

[13] Speakman JR, Talbot DA, Selman C, Snart S, McLaren JS, Redman P, Krol E, Jackson DM, Johnson MS, Brand MD (2004). Uncoupled and surviving: individual mice with high metabolism have greater mitochondrial uncoupling and live longer. Aging Cell.

[14] Speakman JR, Garratt M (2014). Oxidative stress as a cost of reproduction: beyond the simplistic trade-off model. BioEssays.

[15] Speakman JR, Blount JD, Bronikowski AM, Buffenstein R, Isaksson C, Kirkwood TBL, Monaghan P, Ozanne SE, Beaulieu M, Briga M (2015). Oxidative stress and life histories: unresolved issues and current needs. Ecol Evol.

[16] Burda H, Honeycutt RL, Begall S, Locker-Grütjen O, Scharff A (2000). Are naked and common mole-rats eusocial and if so, why?. Behav Ecol Sociobiol.

[17] Bappert M-T, Burda H, Begall S (2012). To mate or not to mate? Mate preference and fidelity in monogamous Ansell’s mole-rats, *Fukomys anselli*, Bathyergidae. Folia Zool.

[18] Dammann P, Burda H (2006). Sexual activity and reproduction delay ageing in a mammal. Curr Biol.

[19] Burda H (1995). Individual recognition and incest avoidance in eusocial common mole-rats rather than reproductive suppression by parents. Experientia.

[20] Schielke CKM, Begall S, Burda H (2012). Reproductive state does not influence activity budgets of eusocial Ansell’s mole-rats, *Fukomys anselli* (Rodentia, Bathyergidae): a study of locomotor activity by means of RFID. Mammal Biol.

[21] Skliba J, Lovy M, Hrouzkova E, Kott O, Okrouhlik J, Sumbera R (2014). Social and environmental influences on daily activity pattern in free-living subterranean rodents: the case of a eusocial bathyergid. J Biol Rhythm.

[22] Henning Y, Vole C, Begall S, Bens M, Broecker-Preuss M, Sahm A, Szafranski K, Burda H, Dammann P (2014). Unusual ratio between free thyroxine and free triiodothyronine in a long-lived mole-rat species with bimodal ageing. PLoS One.

[23] Begall S, Berendes M, Schielke CK, Henning Y, Laghanke M, Scharff A, van Daele P, Burda H (2015). Temperature preferences of African mole-rats (family Bathyergidae). J Therm Biol.

[24] Lighton J (2008). Measuring metabolic rates - a manual for scientists.

[25] Amaratunga D, Ge N (1998). Step-down trend tests for identifying the minimum effective dose. J Biopharm Stat.

[26] Venkatraman E: clinfun: clinical trial design and data analysis functions. R package. R package version 1.0.10 edition; 2015.

[27] Bates D, Machler M, Bolker B, Walker S: lme4: linear mixed-effects models using Eigen and S4. R package version 1.1–8 edition; 2015.

[28] R Core Team (2015). R: A language and environment for statistical computing.

[29] Zelová J, Sumbera R, Sedlácek F, Burda H (2007). Energetics in a solitary subterranean rodent, the silvery mole-rat, *Heliophobius argenteocinereus*, and allometry of RMR in African mole-rats (Bathyergidae). Comp Biochem Physiol A Mol Integr Physiol.

[30] Douhard F, Lemaitre JF, Rauw WM, Friggens NC (2016). Allometric scaling of the elevation of maternal energy intake during lactation. Front Zool.

[31] Zheng G-X, Lin J-T, Zheng W-H, Cao J, Zhao Z-J. Energy intake, oxidative stress and antioxidant in mice during lactation. Zool Res. 2015;36:95–102. 10.13918/j.issn.2095-8137.2015.2.95 PMC4790255 25855228

[32] Schmidt CM, Jarvis JUM, Bennett NC (2013). The long-lived queen: reproduction and longevity in female eusocial Damaraland mole-rats (*Fukomys damarensis*). Afr Zool.

[33] Scantlebury M, Speakman JR, Oosthuizen MK, Roper TJ, Bennett NC (2006). Energetics reveals physiologically distinct castes in a eusocial mammal. Nature.

[34] Keil G, Cummings E, de Magalhães JP (2015). Being cool: how body temperature influences ageing and longevity. Biogerontology.

[35] Douhard F, Lemaître J-F, Rauw WM, Friggens NC: Allometric scaling of the elevation of maternal energy intake during lactation. Front Zool. 2016;13. doi:10.1186/s12983-016-0164-y. 10.1186/s12983-016-0164-y PMC4944469 27418939

[36] Yang DB, Xu YC, Wang DH, Speakman JR (2013). Effects of reproduction on immuno-suppression and oxidative damage, and hence support or otherwise for their roles as mechanisms underpinning life history trade-offs, are tissue and assay dependent. J Exp Biol.

[37] Xu Y-C, Yang D-B, Speakman JR, Wang D-H (2014). Oxidative stress in response to natural and experimentally elevated reproductive effort is tissue dependent. Funct Ecol.

[38] Vaanholt LM, Milne A, Zheng Y, Hambly C, Mitchell SE, Valencak TG, Allison DB, Speakman JR (2016). Oxidative costs of reproduction: Oxidative stress in mice fed standard and low antioxidant diets. Physiol Behav.

[39] Sudyka J, Casasole G, Rutkowska J, Cichoń M (2016). Elevated reproduction does not affect telomere dynamics and oxidative stress. Behav Ecol Sociobiol.

[40] Oklejewicz M, Daan S (2002). Enhanced longevity in tau mutant Syrian hamsters, *Mesocricetus auratus*. J Biol Rhythm.

[41] Echtay KS, Roussel D, St-Pierre J, Jekabsons MB, Cadenas S, Stuart JA, Harper JA, Roebuck SJ, Morrison A, Pickering S (2002). Superoxide activates mitochondrial uncoupling proteins. Nature.

[42] Keipert S, Voigt A, Klaus S (2011). Dietary effects on body composition, glucose metabolism, and longevity are modulated by skeletal muscle mitochondrial uncoupling in mice. Aging Cell.

[43] Salin K, Auer SK, Rudolf AM, Anderson GJ, Cairns AG, Mullen W, Hartley RC, Selman C, Metcalfe NB (2015). Individuals with higher metabolic rates have lower levels of reactive oxygen species in vivo. Biol Lett.

[44] Begall S, Burda H (1998). Reproductive characteristics and growth in the eusocial Zambian Common mole-rat (Cryptomys sp., Bathyergidae). Mammal Biol - Z Säugetierkunde.

[45] Burda H (1989). Reproductive biology (behaviour, breeding, and postnatal development) in subterranean mole-rats, *Cryptomys hottentotus* (Bathyergidae). Mammal Biol - Z Säugetierkunde.

[46] Brand MD (2000). Uncoupling to survive? The role of mitochondrial inefficiency in ageing. Exp Gerontol.

[47] Busiello RA, Savarese S, Lombardi A (2015). Mitochondrial uncoupling proteins and energy metabolism. Front Physiol.

[48] Andziak B, O’Connor TP, Qi W, DeWaal EM, Pierce A, Chaudhuri AR, Van Remmen H, Buffenstein R (2006). High oxidative damage levels in the longest-living rodent, the naked mole-rat. Aging Cell.

[49] Detillion CE, Craft TKS, Glasper ER, Prendergast BJ, DeVries AC (2004). Social facilitation of wound healing. Psychoneuroendocrinology.

